# Supplementary material for: Glucosylceramide Synthase Inhibitors Induce Ceramide Accumulation and Sensitize H3K27 Mutant Diffuse Midline Glioma to Irradiation
Source: Int J Mol Sci. 2023 Jun 8;24(12):9905. doi: 10.3390/ijms24129905 (PMC10298524; doi:10.3390/ijms24129905)
Supplement: Supplementary file 1 [file ijms-24-09905-s001.zip › ijms-2387419-Supplemental Figures.pdf]

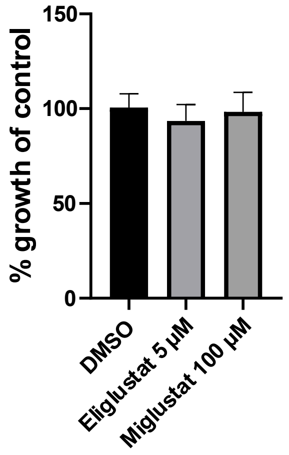

Supplemental Figure S1. Effect of miglustat and eliglustat on cell viability. SF8628 cells were treated with the vehicle (DMSO) or with eliglustat (5 µM) or miglustat (100 µM) for 72h. Results are shown as % growth with respect to untreated cells. The experiments were performed three times. No significant effect on cell viability was observed

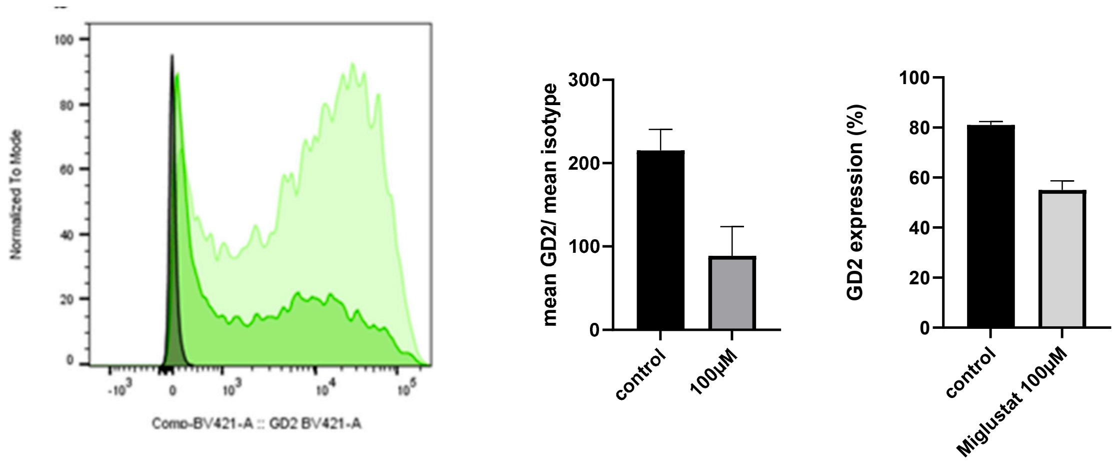

Supplemental Figure S2. Effect of Miglustat on GD2 cell surface expression of H3K27M- mutant DMG cells. SF8628 cells were treated with 100 µM miglustat or mock-treated (control) for 10 days. GD2 expression was analyzed on flow cytometer. The light green histogram shows GD2 expression of mock-treated cells, whereas the dark green histogram shows GD2 expression after miglustat treatment. Isotype control is also illustrated (black histogram). One out of two independent experiments is shown. The bar graphs show the percentage of GD2-positive cells (GD2 expression) and the GD2 intensity (as ratio of mean GD2 to mean of isotype). Mean values of two experiments in % ± SD are shown.

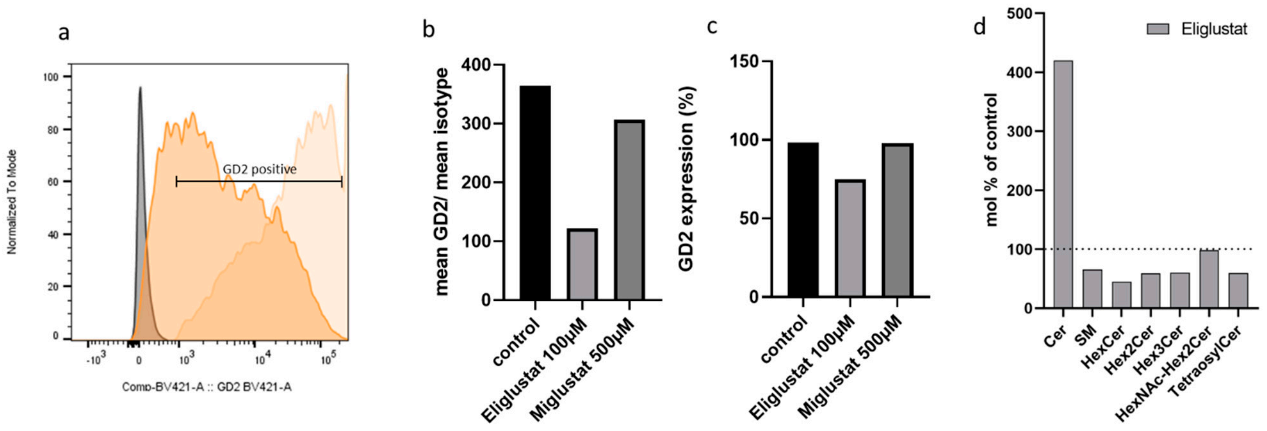

Supplemental Figure S3. GSI inhibit the GSL metabolism in primary H3K27M- mutant DMG cells. The 409 cells were treated with 100 µM eliglustat or 500 µM miglustat or mock-treated (control) for 24h. GD2 expression was analysed by flow cytometry (a). The light orange histogram shows GD2 expression of mock-treated cells, whereas the dark orange histograms show GD2 expression after eliglusat treatment. Isotype control is also illustrated (black histogram). The bar graphs show the proportion of GD2-positive cells (GD2 expression) (c) and the mean of GD2 intensity after treatment with eliglustat (b) respectively. (d) After treatment with 100 µM eliglustat for 24h, sphingolipids were analyzed by mass spectrometry and quantified as mol percent of mock-treated cells (control). The experiments were performed once.

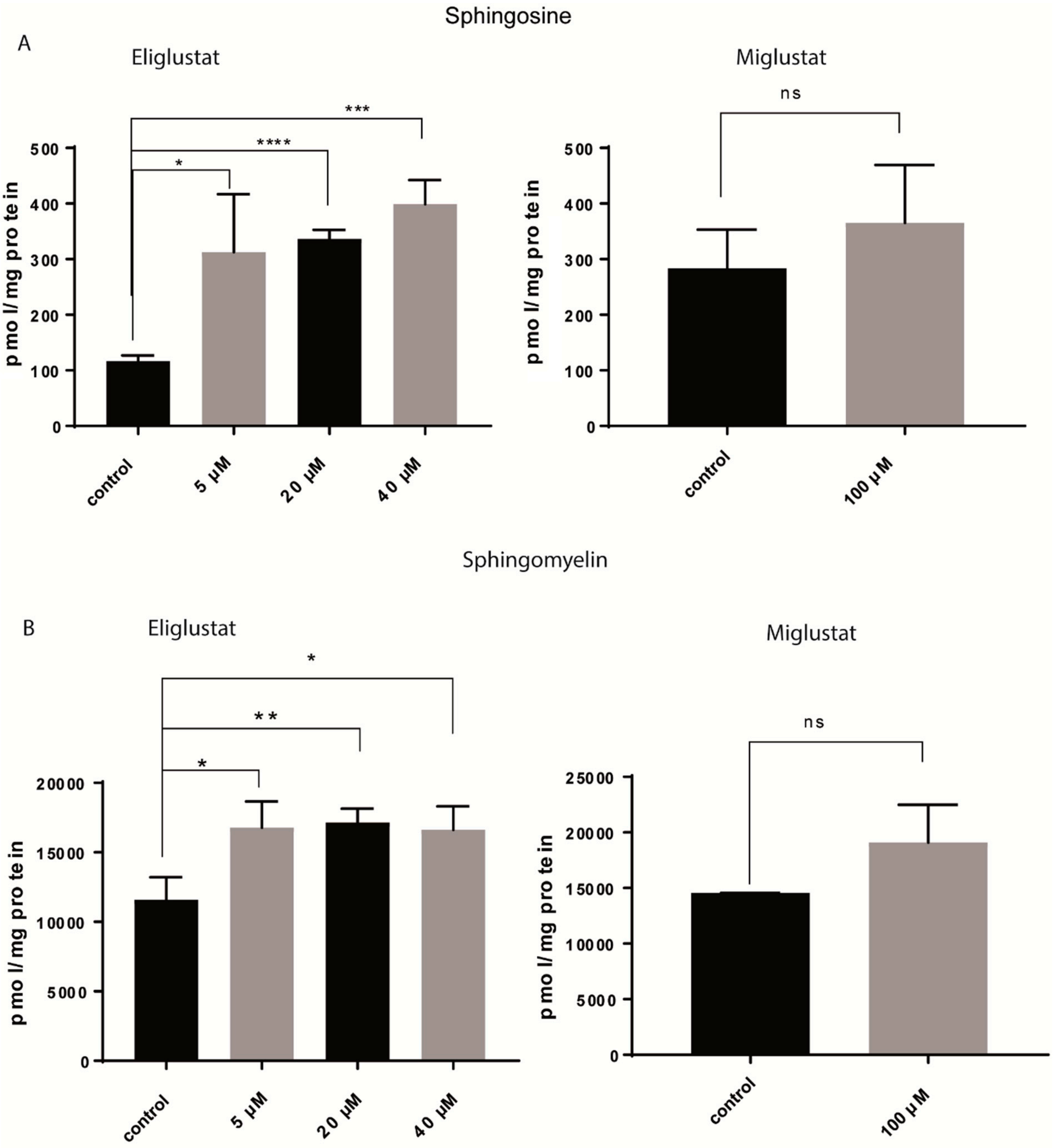

Supplemental Figure S4. Effect of miglustat and eliglustat on sphingosine and sphingomyelin concentration. SF8628 cells were treated with eliglustat at different concentrations for 24 h or with 100 µM miglustat for 72 h. Control refers to the mock-treated cells. Sphingosine (**A**) and sphingomyelin (**B**) were analyzed by mass spectrometry and quantified as pmol/mg protein. Data are shown as means ± SD from three independent experiments. The statistical comparison against the mock-treated samples was performed using student's t-test: *$p<0.05$, **$p<0.01$, ***$p<0.0005$, ****$p<0.0001$, ns: not significant

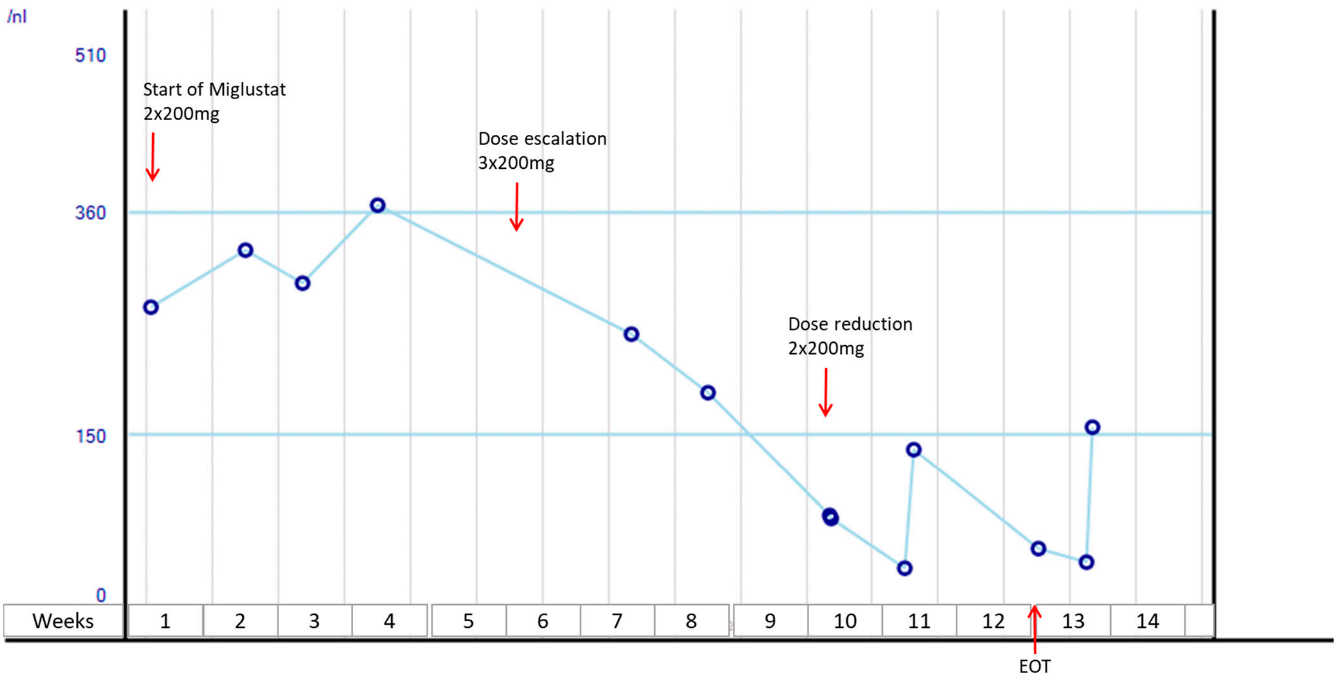

Supplemental Figure S5. Thrombocytes count during treatment with miglustat. Thrombocytes count is shown as number of Thrombocytes per nl. EOT: end of treatment
